# Reliable and Valid Robotic Assessments of Hand Active and Passive Position Sense in Children With Unilateral Cerebral Palsy

**DOI:** 10.3389/fnhum.2022.895080

**Published:** 2022-08-01

**Authors:** Monika Zbytniewska-Mégret, Lisa Decraene, Lisa Mailleux, Lize Kleeren, Christoph M. Kanzler, Roger Gassert, Els Ortibus, Hilde Feys, Olivier Lambercy, Katrijn Klingels

**Affiliations:** ^1^Rehabilitation Engineering Laboratory, Department of Health Sciences and Technology, Institute of Robotics and Intelligent Systems, ETH Zurich, Zurich, Switzerland; ^2^Department of Rehabilitation Sciences, KU Leuven, Leuven, Belgium; ^3^Faculty of Rehabilitation Sciences, Rehabilitation Research Center (REVAL), University of Hasselt, Diepenbeek, Belgium; ^4^Department of Development and Regeneration, KU Leuven, Leuven, Belgium; ^5^Future Health Technologies, Singapore-ETH Centre, Campus for Research Excellence and Technological Enterprise (CREATE), Singapore, Singapore

**Keywords:** cerebral palsy, proprioception, robotics, assessment, hand, rehabilitation

## Abstract

Impaired hand proprioception can lead to difficulties in performing fine motor tasks, thereby affecting activities of daily living. The majority of children with unilateral cerebral palsy (uCP) experience proprioceptive deficits, but accurately quantifying these deficits is challenging due to the lack of sensitive measurement methods. Robot-assisted assessments provide a promising alternative, however, there is a need for solutions that specifically target children and their needs. We propose two novel robotics-based assessments to sensitively evaluate active and passive position sense of the index finger metacarpophalangeal joint in children. We then investigate test-retest reliability and discriminant validity of these assessments in uCP and typically developing children (TDC), and further use the robotic platform to gain first insights into fundamentals of hand proprioception. Both robotic assessments were performed in two sessions with 1-h break in between. In the passive position sense assessment, participant's finger is passively moved by the robot to a randomly selected position, and she/he needs to indicate the perceived finger position on a tablet screen located directly above the hand, so that the vision of the hand is blocked. Active position sense is assessed by asking participants to accurately move their finger to a target position shown on the tablet screen, without visual feedback of the finger position. Ten children with uCP and 10 age-matched TDC were recruited in this study. Test-retest reliability in both populations was good (intraclass correlation coefficients (ICC) >0.79). Proprioceptive error was larger for children with uCP than TDC (passive: 11.49° ± 5.57° vs. 7.46° ± 4.43°, *p* = 0.046; active: 10.17° ± 5.62° vs. 5.34° ± 2.03°, *p* < 0.001), indicating discriminant validity. The active position sense was more accurate than passive, and the scores were not correlated, underlining the need for targeted assessments to comprehensively evaluate proprioception. There was a significant effect of age on passive position sense in TDC but not uCP, possibly linked to disturbed development of proprioceptive acuity in uCP. Overall, the proposed robot-assisted assessments are reliable, valid and a promising alternative to commonly used clinical methods, which could help gain a better understanding of proprioceptive impairments in uCP, facilitating the design of novel therapies.

## Introduction

With a prevalence of 1.5–2.5 per 1,000 live births, cerebral palsy (CP) is the most commonly documented motor impairment in children (Oskoui et al., 2013). It is a non-progressive disorder due to damage of the developing fetal or infant brain (Rosenbaum et al., 2007). In 44% of the children with CP, impairments are predominantly situated on one side of the body (Cans, 2001), mostly in the upper limb (Wiklund and Uvebrant, 2008), which is known as unilateral cerebral palsy (uCP) (Himmelmann and Uvebrant, 2018). Although CP is primarily seen as a motor condition, up to 90% of children with uCP experience somatosensory dysfunctions and show impairments in tactile perception, tactile discrimination, stereognosis and/or proprioception (Bleyenheuft and Gordon, 2013). Proprioception, which has been defined as the ability to perceive body and limb position, orientation and movement, is particularly needed for the execution of fine motor tasks (Gentilucci et al., 1994; Hillier et al., 2015). Since proprioception plays an important role in planning and adapting movements (Hillier et al., 2015), impairment of that modality affects motor control and motor learning in children with CP (Vercher et al., 2003; Robert et al., 2013). Although proprioceptive impairments are most commonly found in the non-dominant hand in children with uCP, it has been shown that in some children, proprioception can also be affected in the dominant hand (Wingert et al., 2009).

Proprioception embodies different modalities, such as active or passive position sense (Hillier et al., 2015; Poitras et al., 2021). Active position sense entails moving the limb to a prespecified position, whereas passive position sense refers to the ability to determine the static position of a body part without moving it, both without relying on vision (Chrysagis et al., 2021). It has been shown that proprioceptive accuracy increases with age (Goble et al., 2005; Marini et al., 2017) and that active position sense becomes more accurate in able-bodied adults than passive position sense (Fuentes and Bastian, 2010). Because of the brain lesion, the effect of age on proprioception modalities might be different in children with uCP, however these trends have not yet been extensively investigated. Gaining a better fundamental understanding of different properties of proprioception in uCP may help targeting motor control deficits and thus improve current rehabilitation techniques.

In order to perform a detailed investigation of proprioceptive deficits and how they are influenced by various factors, such as age and pathological conditions, sensitive assessments are required. Currently, several clinical methods exist in literature to quantify proprioception (Hillier et al., 2015; Poitras et al., 2021). However, it remains challenging to use those clinical assessments, due to their lack of reliability, ordinal scoring and subjectivity (Lincoln et al., 1991). Technology-driven assessment solutions could provide a promising complement to conventional clinical assessments (Scott and Dukelow, 2011; Lambercy et al., 2012; Cappello et al., 2015; Schwarz et al., 2019). Robot-assisted methods are objective (independent of observer judgment), accurate (able to measure exact body position), as well as capable of delivering precise, reproducible stimuli. Moreover, robotic methods allow to investigate several aspects of proprioception, such as active or passive position sense, given that robotic devices can both displace a participant's limb and provide a friction-free environment within which a participant can move while measuring kinematics and kinetics (Kenzie et al., 2017; Semrau et al., 2017; Schwarz et al., 2019). In children with uCP, robotic devices have mainly been used to investigate the passive position sense of the proximal joints of the upper limb (Kuczynski et al., 2016). Nevertheless, proprioceptive deficits may in fact be more pronounced distally, as it has been shown for motor impairments in uCP (Klingels et al., 2012). Currently, to the best of our knowledge, no robotic assessment platform exists to objectively measure the active and passive position sense of the distal joints in children. Therefore, there is a need for a robotic assessment tool of distal position sense, which is adapted to the specific requirements of the CP population, considering their age, visuomotor impairments and engagement in the assessment.

In this paper, we propose robot-assisted assessments of active and passive position sense for children with uCP. It was implemented on an existing one-degree of freedom end-effector robot, ETH MIKE, acting on the index finger metacarpophalangeal (MCP) joint (Zbytniewska et al., 2019). The device can accurately displace participant's finger and sensitively measure its position, velocity and force. The index finger was selected due to its relevance in the majority of activities of daily living (Dollar, 2014), while focusing on a single joint simplifies the technology and increases clinical usability. The passive position sense assessment has previously been introduced and validated in stroke patients (Zbytniewska et al., 2021). For the purpose of this work, the active position sense assessment was developed and implemented on the same platform. Furthermore, hardware and software were specifically adapted for children to keep them motivated and engaged throughout the assessment.

The aim of this study was to investigate the test-retest reliability and discriminant validity of the robotic assessments of position sense in children with uCP and typically developing children (TDC), as well as to use that platform to gain insights into fundamentals of finger proprioception, among others, the differences between active and passive position sense. We hypothesized that the proposed assessments are reliable, given that they are based on advanced sensor technology rather than observer judgment, and that they are able to discriminate between children with uCP and TDC, due to the sensitivity of the measure and the lack of ceiling/floor effects. Finally, we hypothesized that TDC would have worse passive than active position sense accuracy, as reported in able-bodies adults (Fuentes and Bastian, 2010). The accuracy may additionally be age-dependent (Marini et al., 2017), while this relationship would be disrupted and more variable in children with uCP. This work aspires to enrich the field of pediatric neurorehabilitation by proposing a novel technological solution to the longstanding challenge of accurate and objective quantification of proprioceptive impairments in children with uCP.

## Materials and Methods

### Participants

Children with uCP aged 7–15 years old were recruited from the CP reference center of the University Hospitals Leuven. As inclusion criteria, children had to have a House Functional Classification Score ≥ 4 (House, 1981), indicating the ability to hold an object with the non-dominant hand, and have sufficient cognitive capacity to understand and follow instructions. Participants were excluded when they received (1) botulinum toxin injections in the upper limb 6 months prior to the assessment or (2) surgery of the upper limb 2 years prior to the assessment. Moreover, age-matched TDC without a neurological disorder or musculoskeletal problems of the upper limb were recruited. This study was approved by the Ethical Committee of the University Hospitals Leuven (S62906) and was performed in accordance with the Declaration of Helsinki. The participant's parents signed an informed consent form, and children older than 12 years additionally assented to participate. General characteristics of the participants were collected from their medical file. These characteristics included the age, gender, side of unilateral CP and the level of the Manual Ability Classification System (MACS), providing general information about the children's manual ability (Eliasson et al., 2006).

### Robot-Assisted Assessments

#### Apparatus

**Figure 1:**
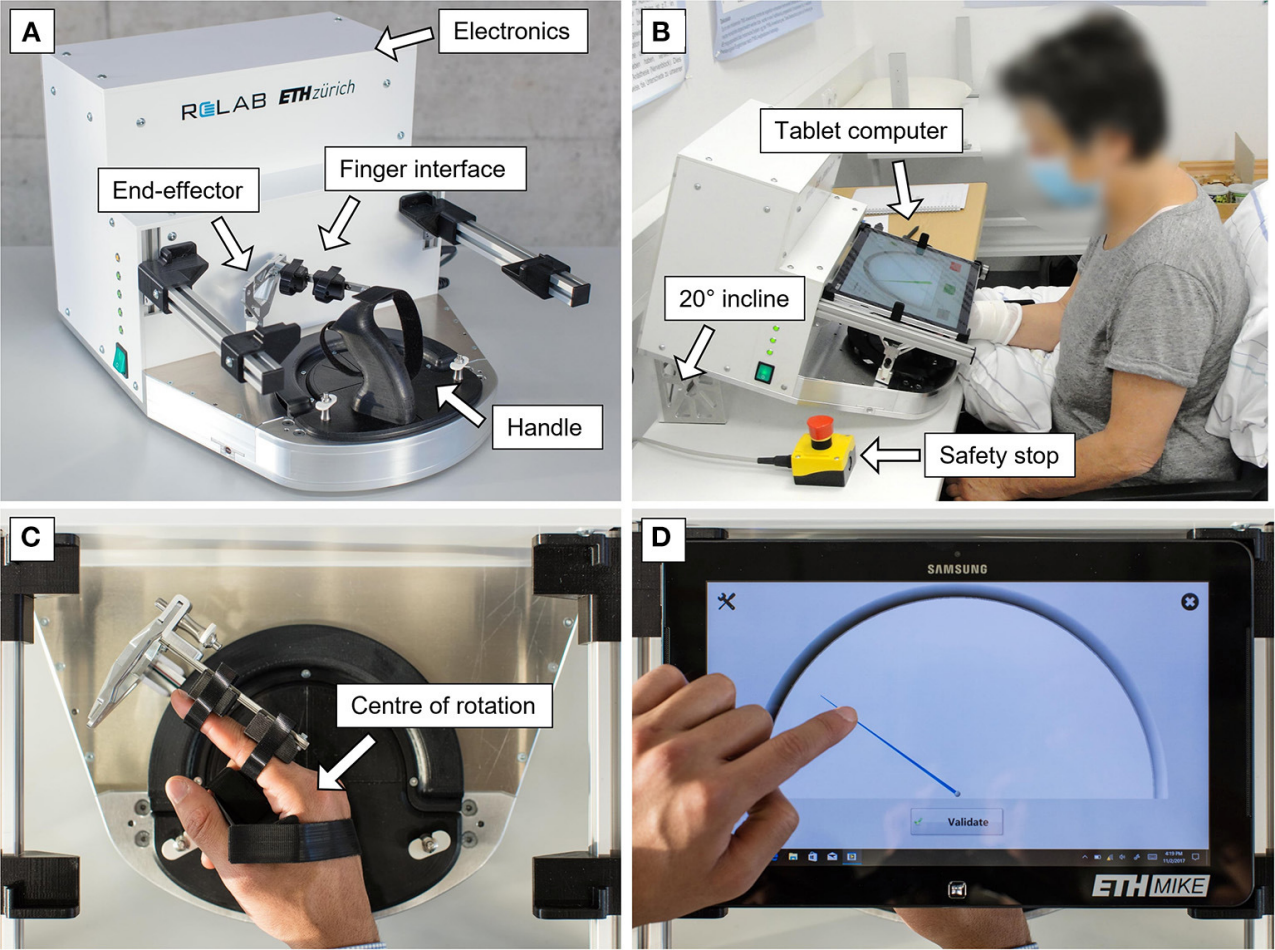
ETH MIKE robotic device for the assessment of finger proprioception. **(A)** The components of the platform interfacing with the user are an end-effector, a finger attachment mechanism and an exchangeable 3D-printed handle. In the white box, all electronic components are located (including an actuator, which generates end-effector movement through a cable transmission system, for more details see Zbytniewska et al., 2019). **(B)** To avoid parallax error, the device is inclined at 20° and participants are seated directly above the tablet computer screen. This image displays an adult participant using the device version based on which the adaptations for children were made. **(C)** The center of the end-effector rotation occurs around the MCP joint of the index finger. **(D)** A simple GUI displays the passive position sense assessment. This GUI is a previous version for adults, which will be adapted to suit children's needs.

The ETH MIKE (Motor Impairment and Kinesthetic Evaluation) is a one degree-of-freedom end-effector robot, which can provide well-controlled stimuli to the index finger in the form of a displacement or a perturbation, while sensitively measuring the finger's response (position, velocity, and force) (Zbytniewska et al., 2019, 2021). The end-effector of the robot is aligned so that the index finger can comfortably rotate around the MCP joint (Figure 1). The actuator is located, together with an incremental encoder and a tachometer, away from the end-effector in an electronics box, and the end-effector rotation is performed through a cable transmission system. During experiments, participants are seated in front of the device with their elbow resting on an armrest. The hand is positioned by grasping a 3D-printed handle (which can easily be exchanged for a left or right-hand version). The index finger is inserted into a finger interface connected to the end-effector, composed of a metal guide, 3D-printed pads, and Velcro straps. A tablet computer displaying the Graphical User Interface (GUI) is located directly above the hand in order to cover participants' vision of their hand, thereby ensuring that they are relying on their proprioceptive abilities to complete the assessments. The GUI displays the assessment interface and allows to create an anonymized participant login, for which basic demographic information is stored (i.e., age, gender, handedness and affected side if applicable). To avoid parallax errors, participants need to be looking straight at the tablet screen during an assessment, therefore the device is inclined at 20° using a designated metallic platform. For safety, there is an emergency stop button placed within close reach of the experimenter and the user, which can disconnect the device from the power supply.

#### Assessment Tasks

##### Passive Position Sense

**Figure 2:**
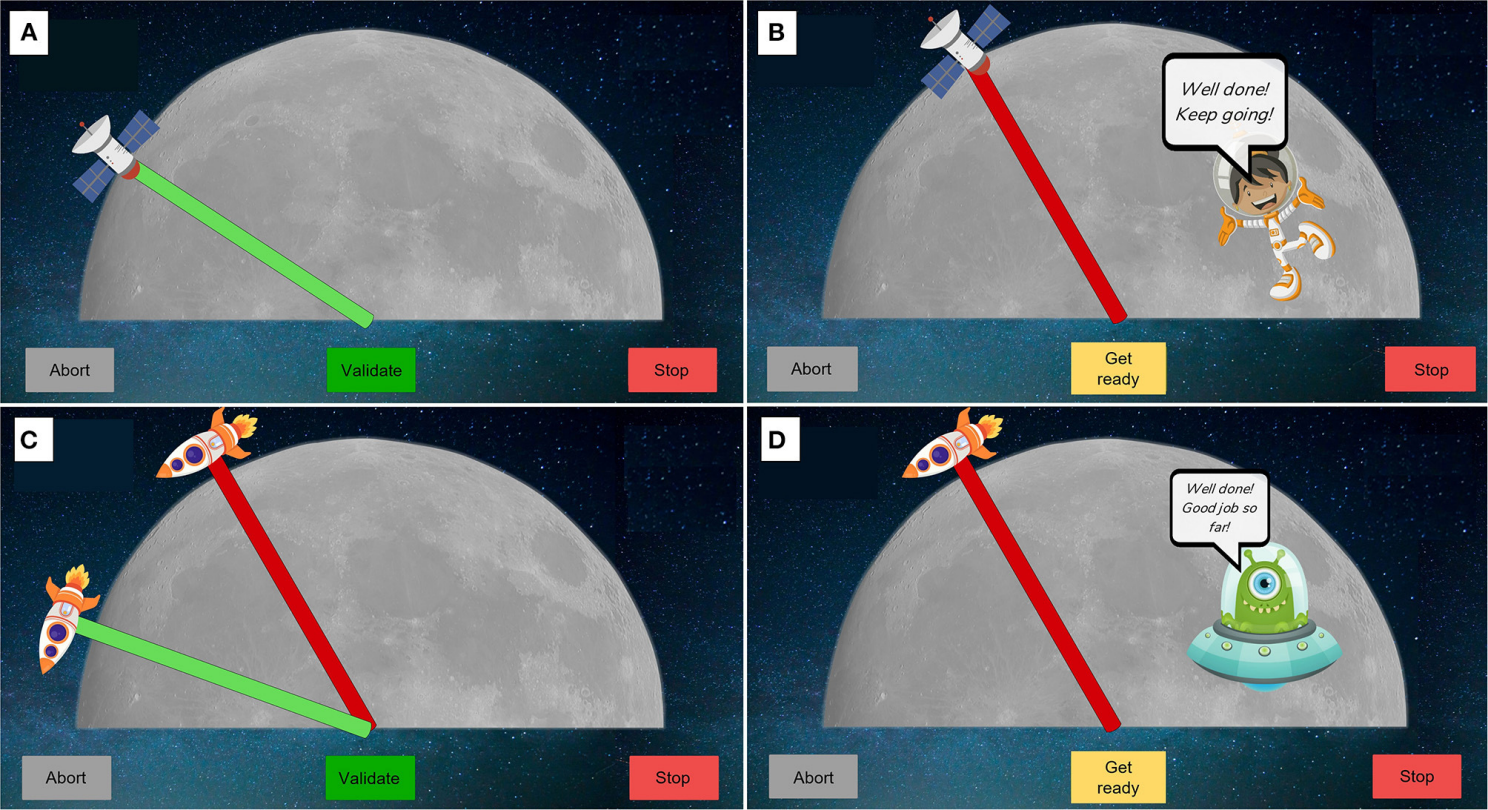
Visualization of assessments interfaces. **(A,B)** Passive position sense assessment. Participants need to indicate using the green needle with a satellite, their perceived finger position. The finger is first moved to the starting position (red) by the robot and then to a position in flexion. **(C,D)** Active position sense assessment. Participants need to move their finger as accurately as possible from a starting position (red needle) to the position of the green needle. The green needle stays visible until participant clicks “validate” button to proceed to next trial, when his/her finger is moved to the starting position. An encouraging character is displayed twice per session when the finger is moved to the starting position **(B,D)**. A “abort” and emergency stop buttons are always displayed on the GUI to allow to quit the assessment if needed.

It is assessed with a Gauge Position Matching task that has been described in detail in previous work (Rinderknecht et al., 2016; Zbytniewska et al., 2021). For the purpose of this study the task protocol was kept identical as in previous studies involving the Gauge Position Matching task on the ETH MIKE to allow for comparability between different populations (Zbytniewska et al., 2021). In brief, the index finger is moved by the robot from a starting position (red needle, Figure 2) to one out of 11 predefined positions (integer values [10–30]° in flexion from the starting position, every 2°). The starting position is at a neutral MCP joint location (0° in flexion, 30° from the middle of the device's workspace). The actual finger position is covered by the tablet screen, hence visual feedback is not provided during the assessment. Participants need to indicate the perceived finger position on the screen above using the other (non-tested) hand by dragging a green gauge needle and pressing a button “validate” once they are satisfied with the indicated position. In case a participant is not able to accurately operate the tablet with their non-dominant hand, the experimenter helps by moving the needle slowly until participant says “stop.” There is a practice round before the assessment, consisting of six trials. Among the six trials, the first three trials display visual feedback in the form of a blue needle indicating the real-time finger position, while the other three are in absence of visual feedback (i.e., as in the assessment condition). These practice trials are not included in the data analyses and serve the purpose of familiarization with the assessment protocol. After the practice, the assessment task starts, which is verbally communicated to the participants. The primary outcome measure is the mean absolute error (AE) between the actual and the indicated position across 11 trials.

##### Active Position Sense

This task was specifically developed for the purpose of this study and provides an important complement to the existing battery of assessments of the ETH MIKE (Zbytniewska et al., 2021). It requires participants to actively move their finger without any assistance from the robot. The aim is to move the finger from a starting position (red needle, Figure 2) to a target (green needle) as accurately as possible, but without visual feedback of the finger position. There are no time constraints and once ready, participants can click the “validate” button to proceed to the next trial. In this study, there were two possible targets, one at 20° flexion and the other at 40° flexion of the MCP joint. Each assessment is preceded by four practice trials, the first two of which display visual feedback of the real-time finger position in the form of a blue needle. These practice trials help with task familiarization and are not included in the data analysis. Participants are then informed when the assessment starts. The assessment consists of 10 trials, without visual feedback, including five trials at 20° flexion and five at 40° flexion in random order. The outcome measure is the mean absolute error (AE) between the actual target position and the position of the finger across all 10 trials.

### Adaptations for Children

**Figure 3:**
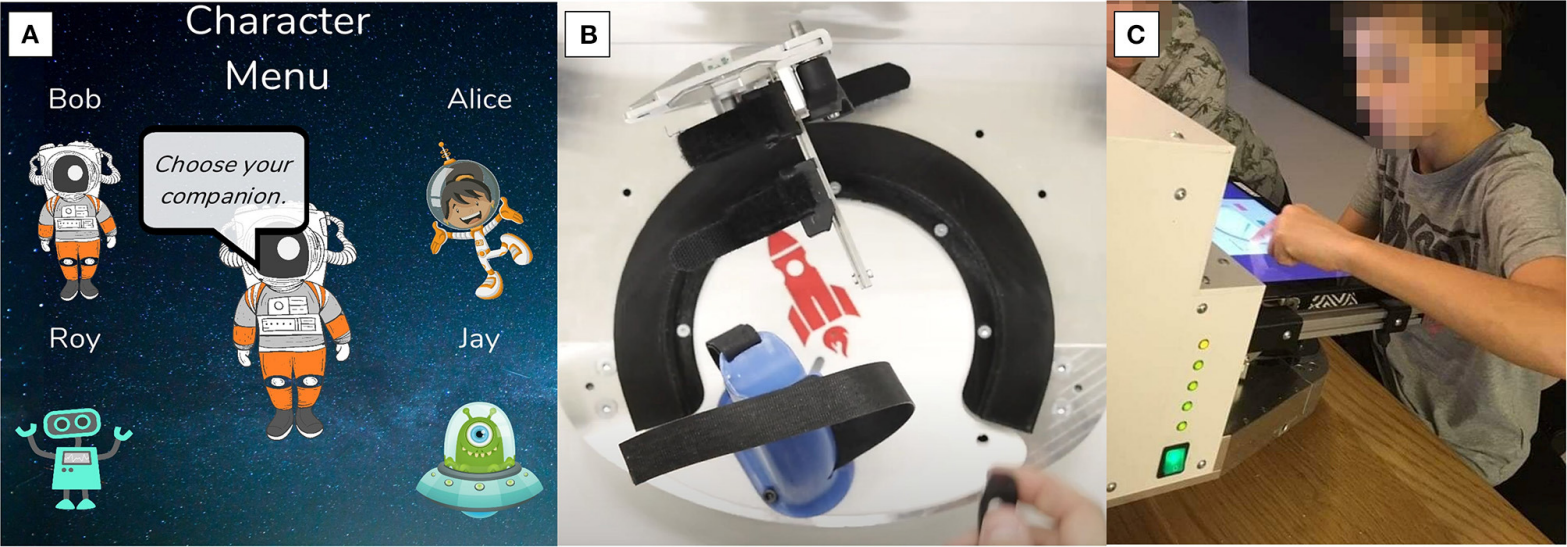
Adaptation of the GUI and of the handles to make the assessment platform more suitable for children. **(A)** The GUI was transformed into a space-theme. At the start of the assessment, children can choose a character that then provides instructions and encouraging messages throughout the assessment. **(B)** The handle was designed with brighter colors and an image of a rocket to match the overall theme. The handle position was made adaptable for different hand sizes. **(C)** A child interacting with the GUI completing the passive position sense assessment.

The original version of the hand interface and software developed for adults (Zbytniewska et al., 2019, 2021) was adapted to make it more suitable for use with children. The adaptations were made based on literature recommendations, feedback from clinicians who had experience working with children with CP as well as feedback from children themselves (Taslim et al., 2009; Weightman et al., 2010). Design requirements were defined considering specific needs of CP, e.g., related to sufficient color contrasts given that visual impairments are common in children with CP (Striber et al., 2019). Firstly, the GUI was adapted from a simple gauge interface (Figure 1D) into a space-themed environment (Figures 2, 3). At the beginning of the assessment, children choose a character that is shown on the screen and provides a standardized encouraging dialogue and instructions, while not providing any information about the performance to ensure no learning is possible during the assessment. This adds a personal element to the assessment experience since children get to choose the character they prefer. Furthermore, the simple gauge was changed into a half-moon and the gauge needle into a satellite or rocket, adding elements of gamification to the assessment (e.g., match the location of the satellite with the tip of your index finger). Finally, the handle was modified to have more appealing colors and an image of a rocket to match the overall assessment theme. The upper part of the handle was made movable on the baseplate to allow the MCP joint to be shifted up for smaller hands ensuring correct alignment with the center of rotation (Figure 3B).

### Study Protocol

This study consisted of two sessions with a 1-h break in between to investigate test-retest reliability. Each session lasted up to 30 min [5 min setup, 10 min for both assessments of the dominant hand (see details of the two assessment tasks in Section Robot-assisted Assessments), 10 min for the non-dominant hand, 5 min as back-up time]. Instructions and encouragement were given by the same experienced therapist during both sessions, following a standardized procedure to ensure consistency in the test and retest administration. The standardized procedure contained written instructions on how to explain the assessments to the participants. If any adjustments were made throughout the testing, such as chair positioning or handle adaptation, the precise configuration was maintained throughout the retest assessment to ensure reproducibility. In both sessions, each task was performed first with the dominant, followed by the non-dominant hand, always starting with the passive position sense task.

### Data Analysis

The descriptive statistics are reported as mean and standard deviation. The statistical analysis and evaluation of the metrics was inspired from a previously established framework for validating digital health metrics (Kanzler et al., 2020). To evaluate test-retest reliability, intraclass correlation coefficient ICC(A,k) and their 95% confidence intervals were calculated based on a two-way mixed-effects model, which considers the degree of absolute agreement for measurements that are averages based on k independent trials made within a fixed time interval (McGraw and Wong, 1996; de Vet et al., 2006). Moreover, the smallest real difference (SRD) and SRD% with respect to the range across all trials were calculated (de Vet et al., 2006; Kanzler et al., 2020). An acceptable value for ICC has previously been defined as above 0.70, between 0.75 and 0.90 as good and above 0.90 as excellent, while for SRD% at 30.3% (Koo and Li, 2016; Prinsen et al., 2018; Kanzler et al., 2020). Learning effects were calculated as the mean difference between test and retest, normalized to the range of observed values (in %). Metrics within the range [−6.35%, 6.35%] were considered not to have strong learning effects (Kanzler et al., 2020). To compare between TDC and uCP groups (discriminant validity analysis), between the modalities of proprioception, as well as to evaluate the effect of personal factors (hand dominance, age) on the task scores, a linear mixed-effect model was implemented (Bolker et al., 2009; Kanzler et al., 2020), using data from both test and retest. Population (TDC or uCP), hand (dominant or non-dominant), proprioceptive modality (active or passive position sense), age and session (test or retest) were considered as fixed effects in the model. Additionally, a subject-specific random effect was added to account for possible intra-subject dependencies arising from including both tested body sides and both test sessions for each subject (Kanzler et al., 2020). The full model is summarized below.


(1) $$\begin{array}{l} \begin{array}{ccc} y_{j} & = & \;\beta_{0}+\;\beta_{1}population_{j}+\;\beta_{2}hand_{j}+\beta_{3}modality_{j}+\beta_{4}age_{j} \end{array}\\ \begin{array}{c} \;+\beta_{5}session_{j}+W_{j}+\epsilon \end{array} \end{array}$$

*y*_*j*_, value of AE of subject *j*; β, model parameters; *W*_*j*_, subject-specific intercept; ϵ, residual error.

A sub-analysis was also performed, in which a separate model was built for active and passive position sense, with the objective of obtaining a separate analysis of the difference between children with uCP and TDC for each proprioceptive modality. Another sub-analysis was performed aiming at a more detailed comparison of proprioceptive modalities, in which a separate model was built for each population. Lastly, the influence of personal factors was additionally analyzed in separate models per population and per modality (4 models: active position sense in uCP, passive position sense in uCP, active position sense in TDC, passive position sense in TDC), in this case keeping age, hand and session as fixed effects and subject-specific effect as a random effect. A Box–Cox transformation was applied to the outcome measure (AE) to correct for heteroscedasticity, as subjectively perceived through non-normally distributed model residuals in quantile–quantile plots (Box and Cox, 1964; Kanzler et al., 2020). Additionally, Spearman's rank correlation (ρ_s_) was used for correlation analysis, including test data only to avoid the bias due to possible intrinsic correlations of test-retest data, and considering sample size requirements for the choice of the correlation type (Bonett and Wright, 2000). The significance level was set to 0.05. Data analysis was conducted in MATLAB R2019a.

## Results

### Participants

In total, 10 children with uCP (mean age 11y6m ± 2y11m, four males, seven right-hand dominant, MACS Level I (*N* = 5) and II (*N* = 5)) and 10 age-matched TDC (mean age 11y7m ± 2y3m, six males, eight right-handed) were included in this study. Detailed table with information about each participant is available in Supplementary Material.

### Test-Retest Reliability

**Figure 4:**
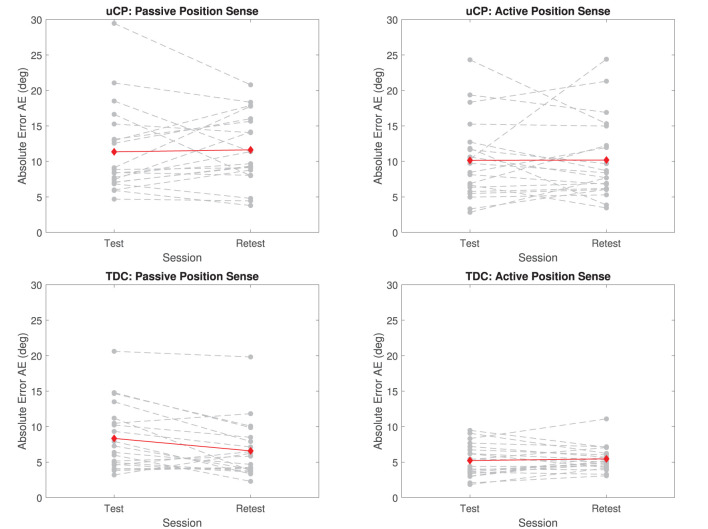
Test-retest reliability in uCP and TDC for passive and active position sense. Gray dashed lines are individual participant's data the red line indicates the mean across all participants at test and retest. Both hands are considered together in the figure (dominant and non-dominant hand, hence *N* = 20).

Test-retest reliability in both populations was good to excellent. Specifically, ICC(A,k) was equal to 0.85 and 0.84 for passive and active position sense in children with uCP, 0.79 and 0.92 in TDC, respectively. The SRD% was 12.91–20.24% of the range of observed values. Learning effects were negligible, ranging from −0.07 to 2.15% (Table 1, Figure 4). In total six children with uCP and one TDC showed higher test-retest variations (difference between test and retest above mean TDC score on the given test). There was no clear trend of specific clinical characteristics that explained why those participants showed higher variability (e.g., among those six children with uCP, three had MACS = 1 and three MACS = 2, age ranged from 7 to 14 years, which corresponds to the range of all included subjects).

**Table 1:** Results of test-retest reliability in uCP and TDC for passive and active position sense assessments.

	**uCP**	**uCP**	**TDC**	**TDC**
	**Passive position sense**	**Active position sense**	**Passive position sense**	**Active position sense**
Absolute Error (°) Test (*N* = 20)	11.35 ± 6.24	10.15 ± 5.64	8.34 ± 4.70	5.22 ± 2.30
Absolute Error (°) Retest (*N* = 20)	11.63 ± 4.97	10.19 ± 5.75	6.58 ± 4.06	5.46 ± 1.77
ICC (CI)	0.85 (0.81–0.89)	0.84 (0.79–0.88)	0.79 (0.72–0.84)	0.92 (0.89–0.94)
SRD (°)	15.42	12.88	12.92	5.31
SRD%	17.83	20.24	15.87	12.91
Learning effects (%)	−0.33	−0.07	2.15	−0.58

*AE, Absolute Error; STD, standard deviation; ICC, intraclass correlation coefficient (A,k); CI, confidence interval; SRD, smallest real difference; SRD%, smallest real difference as a % of observable values. The results for active and passive position sense are calculated considering both hands together (hence N = 20)*.

### Discriminant Validity

**Figure 5:**
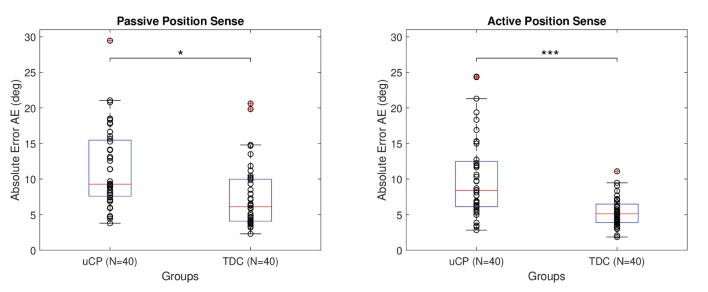
Comparison between uCP and TDC in active and passive proprioceptive absolute error. The stars indicate the following significance: * < 0.05 and *** < 0.001. Data from both hands and both test sessions are considered together in the figure, hence *N* = 40. The smaller the absolute error AE the better the performance (higher acuity).

Absolute error for passive and active position sense was larger for children with uCP than TDC considering both dominant and non-dominant hands together. Namely, passive position sense AE was equal to 11.49° ± 5.57° in uCP and to 7.46° ± 4.43° in TDC, while active position sense AE was equal to 10.17° ± 5.62° in uCP and 5.34° ± 2.03° in TDC, as shown in Table 1. The difference between children with uCP and TDC was statistically significant according to the linear mixed effect model considering all effects together (*R*^2^ = 0.46, *p* < 0.001), as well as according to the models made for each proprioceptive modality separately. The values of the model considering passive position sense only were *R*^2^ = 0.70, *p* = 0.008 and *R*^2^ = 0.41, *p* < 0.001 for the model including active position sense. The differences between uCP and TDC in active and passive position sense AE are shown in Figure 5.

### Comparison Between the Modalities of Proprioception

**Figure 6:**
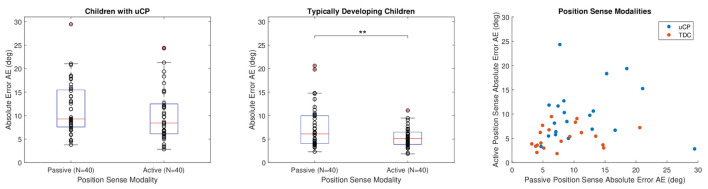
Comparison between passive and active position sense for uCP and TDC. The stars indicate the following significance: ** < 0.01. Data from both hands and test sessions are considered together in the box plots (*N* = 40). In the correlation plot, data from test session only and for both hands is considered (*N* = 20 for each group).

Active position sense absolute error was smaller than passive for both children with uCP and TDC. The difference between active and passive position sense was significant when considering all factors together according to the values of the linear mixed-effect model *R*^2^ = 0.46, *p* = 0.002. However, when considering children with uCP and TDC separately, this effect was significant for TDC (*R*^2^ = 0.33, *p* = 0.005) but not for children with uCP (*R*^2^ = 0.29, *p* = 0.106), as visualized in Figure 6. These two modalities of proprioception were not significantly correlated in TDC according to Spearman correlation ρ_s_ = 0.29; *p* = 0.22; *N* = 20, nor in uCP, where ρ_s_ = 0.24; *p* = 0.31, *N* = 20.

### The Effect of Age on Position Sense

**Figure 7:**
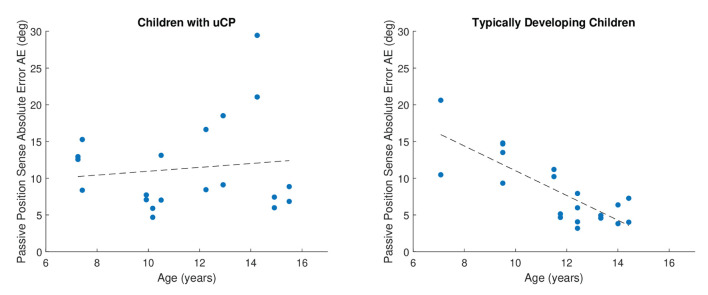
Visualization of the effect of age on passive position sense. A clear trend and a statistically significant effect of age on passive position sense was found in TDC (*N* = 20) but not in children with uCP (*N* = 20) children. Data from both hands are considered together in the figure. Younger TDC tended to have higher passive position sense errors than older ones, which means that proprioceptive performance increased with age. The lower the Absolute Error the better.

The effect of age on position sense was significant according to the linear mixed-effect model considering all factors, *R*^2^ = 0.46, *p* = 0.021. When analyzing children with uCP and TDC separately, position sense absolute error was influenced by age in TDC (*R*^2^ = 0.33, *p* < 0.001), but not in children with uCP (*R*^2^ = 0.29, *p* = 0.673). Further, within the two modalities of proprioception in TDC, the effect of age was significant for passive (*R*^2^ = 0.60, *p* < 0.001) but not for active (*R*^2^ = 0.37, *p* = 0.176) position sense. There was a significant negative correlation between passive position sense and age in TDC, as reported by Spearman correlation ρ_s_ = −0.76; *p* < 0.001, but not in uCP, where ρ_s_ = −0.12; *p* = 0.612, as shown in Figure 7.

### Comparison Between Dominant and Non-dominant Hand

**Figure 8:**
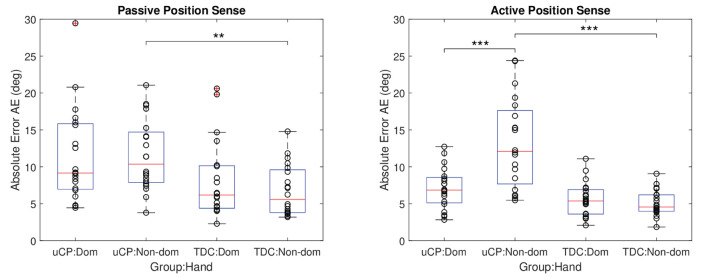
Comparison between hands for active and passive position sense. Data from both test sessions are considered together for each group (*N* = 20). Dom, dominant; non-dom, non-dominant hand. The stars indicate the following significance: ** < 0.01 and *** < 0.001.

Hand dominance was not significantly affecting position sense when considering all effects, as reported through model values *R*^2^ = 0.46, *p* = 0.104. However, a further sub-analysis using the model considering each population separately revealed that there was a significant effect of hand dominance in children with uCP (*R*^2^ = 0.29, *p* < 0.001) but not in TDC (*R*^2^ = 0.33, *p* = 0.247). Further considering each proprioceptive modality separately in children with uCP, performing the active position sense assessment with the dominant hand led to lower absolute errors than when performed with the non-dominant hand, which was not the case for passive position sense, see Table 2 for details. The comparison between body sides and between groups is summarized in Figure 8.

**Table 2:** Comparison of dominant and non-dominant hands in uCP and TDC.

	**Passive position sense**		**Active position sense**	
	**uCP (*N* = 20)**	**TDC (*N* = 20)**	**uCP (*N* = 20)**	**TDC (*N* = 20)**
Dominant	11.45° ± 6.38°	8.15° ± 5.24°	7.10° ± 2.78°	5.62° ± 2.28°
Non-dominant	11.53° ± 4.80°	6.77° ± 3.44°	13.24° ± 6.11°	5.06° ± 1.75°
Linear mixed effect model *R*^2^ and *p*-value	*R*^2^ = 0.64, *p* = 0.56	*R*^2^ = 0.60, *p* = 0.24	*R*^2^ = 0.31, *p* = 0.0008	*R*^2^ = 0.37, *p* = 0.33

*Data from both test sessions are included*.

## Discussion

The aim of this study was to evaluate test-retest reliability and validity of a robot-assisted assessment of active and passive position sense in children with uCP and TDC, as well as to gain first insights into behavioral properties of finger proprioception in children. We found that both measures of active and passive position sense were reliable in children with uCP and TDC and able to discriminate between these two populations. TDC performed significantly more accurately in active compared to passive position sense assessment. Further, we found that the correlation between active and passive position sense, as measured with our robotic assessment, was non-significant in children with uCP and TDC. This suggests these two components of proprioception have some unique properties and require separate evaluation. Moreover, children with uCP were more severely affected in active, but not in passive position sense in their non-dominant hand. Finally, we found a significant effect of age on passive position sense in TDC but not in children with uCP. Overall, the robotic assessment method used in this study is promising to bring new insights into active and passive finger position sense in children with uCP, which could contribute to answering important physiological questions related to proprioception.

This work is novel, as it is one of the few studies investigating proprioception at the level of distal joints of the upper limb using robotics, and the first to focus specifically on finger proprioception in children with uCP, since existing studies either targeted more proximal upper limb joints or were aimed at different populations (Kuczynski et al., 2016; Marini et al., 2017; Rinderknecht et al., 2018; Ingemanson et al., 2019). Proprioception at the level of the fingers as investigated in this study is necessary for many activities of daily living involving manual dexterity, such as using cutlery or as tying one's shoelaces, which is frequently affected in children with uCP (Jones, 1996; Bleyenheuft and Gordon, 2013). In our approach we specifically consider index finger metacarpophalangeal joint, which is relevant for many ADLs (Dollar, 2014) and allows to reduce the complexity of the assessment itself, making it more clinically usable. Information from a single joint should be to some degree representative for the whole hand, since it has been previously reported that there is a high level of agreement in somatosensory deficits between neighboring joints (Connell et al., 2008; Busse and Tyson, 2009). Moreover, the presented assessment method is unique at distinctly evaluating finger passive and active position sense in one device, which makes comparing them easier, but has only been done for position sense and kinesthesia (Semrau et al., 2019). The proposed finger position sense assessments could be used in the future to investigate the relationship between distal and proximal proprioception, as well as to better understand its importance for ADLs. These insights could further help in developing relevant therapies targeting somatosensory impairments of the upper limb, with the overreaching objective of improving performance in activities of daily living.

### Clinimetric Properties: Test-Retest Reliability and Discriminant Validity

It is important to determine clinimetric properties of any newly introduced assessment before its extensive use, especially in longitudinal studies. Lack of such evaluations has been shown as one of the main burdens of clinical implementations of novel technological solutions (Schwarz et al., 2019; Shirota et al., 2019). Previous work has often assumed reliability of an assessment method that has been tested in another population, while it is important to conduct such evaluation in each target population, given pathological differences between disorders or age differences (Kuczynski et al., 2016; Marini et al., 2017). In this work we showed that assessment of passive and active proprioception are reliable, as measured by good ICC above 0.75, measurement error below 30.3% of the range of observable of values and negligible learning effects, specifically within the previously defined range [−6.35, 6.35]% (Kanzler et al., 2020). In that sense, the reliability results in children with uCP are comparable to our previous findings in adult stroke using the same apparatus to evaluate passive position sense (ICC = 0.90 in adult stroke, 0.85 in children with uCP) (Zbytniewska et al., 2021). It is an important finding that good reliability could be demonstrated also in children, as it has previously been shown challenging to achieve satisfying reliability of proprioceptive assessments (Lincoln et al., 1991; Rinderknecht et al., 2018). Some test-retest variability could have been expected in children with uCP, especially in the active proprioception task involving motor function, as motor execution variability has been shown to be correlated with the severity of motor impairment in CP (Cheng et al., 2021). Although this study only included individuals with object handling capabilities (MACS Level I–II), considerable variability in motor impairments is still expected within this group. Likely due to the restricted workspace of the proposed assessments to one-degree of freedom and simple task instructions, the sources of performance variability could be reduced, resulting in good reliability. Moreover, the objective measurement capabilities of the robotic technology remove a potential source of intra-subject variability coming from observer-based judgement, which is one of the main challenges of the clinical scales (Lincoln et al., 1991). Sensitivity of the sensor-based scale leads to higher inter-subject variability and lack of ceiling/floor effects, another factor contributing to satisfying reliability results (Schwarz et al., 2019; Kanzler et al., 2020).

Next to this, discriminant validity was determined, as there was a significant difference in both active and passive modalities of position sense between children with uCP and TDC, especially on the non-dominant hand. This indicates that the measures are capable of distinguishing between impaired and normal position sense and confirms previous work showing that on average children with uCP have larger proprioceptive errors than TDC (Wingert et al., 2009; Kuczynski et al., 2016). Further, the passive position sense results of TDC obtained in this study are comparable to results in older adults obtained in a previous study [AE = 6.77° ± 3.44° in 10 TDC, AE = 5.21° ± 2.71° in 31 older adults aged 66.87 ± 7.92 years, non-dominant hand in both groups (Zbytniewska et al., 2021)]. This shows that despite some modifications of the system to make it more suitable for children, the robustness of the measurement was not affected.

Taken together, the proposed robotic assessments of hand active and passive position sense have good clinimetric properties, making them suitable for future comprehensive, cross-sectional, and longitudinal evaluations of proprioception.

### Using Robotic Metrics to Better Understand Proprioception in Children With uCP and TDC

In the comparison between the two modalities of proprioception, we found that in TDC, active position sense error was significantly smaller than passive position sense error. These results confirm previous work in able-bodied adults showing that self-generated movement provides more accurate proprioceptive information than when a body part is displaced by an external source (Fuentes and Bastian, 2010). This phenomenon might be explained by the fact that active movements generate additional position information from efference copies of motor commands and alpha-gamma motor neuron coactivation, which leads to higher proprioceptive accuracy (Laufer et al., 2001; Gandevia et al., 2006; Fuentes and Bastian, 2010). Moreover, active and passive position sense were not correlated in TDC nor in children with uCP. Indeed, both of these proprioception modalities rely on proprioceptive afferent information to estimate one's limb position, however in the active movement there is additionally kinematic information available from the central estimate due to the operations of an internal forward model (Chokron et al., 2004; Proske and Gandevia, 2012; Capaday et al., 2013). That additional information might lead to some level of dissociation between these proprioception modalities. Further, in case of uCP, the processes responsible for active and passive position sense might be differentially affected for some individuals. First, the size of the lesion may play a role, as it has been shown that in adult stroke, a larger lesion resulted in impairments in more proprioceptive modalities, namely passive position sense and kinesthesia (or the ability to recognize movement), compared to a smaller lesion of which only passive position sense was affected (Semrau et al., 2019). We could hypothesize that the extent of lesion could also play an important role on the different sub-modalities of proprioception in children with uCP, resulting in differentially impaired passive and active position sense. Second, the underlying motor impairments, especially prominent on the more affected side of children with uCP, may also play a role as the active proprioception task requires to selectively move the finger to the target position, which forms an additional challenge for children with uCP. This could be confirmed by our results showing a significant difference between hands for the active position sense assessment, indicating worse active position sense in the non-dominant hand in children with uCP. Although the participating children generally were able to handle objects (MACS Level I–II), mild to moderate motor impairments could still be present, such as spasticity, lack of selective movements, muscle shortness or limited range of motion (Arner et al., 2008; Klingels et al., 2012). The exact influence of various aspects of motor impairments on the performance in the active position sense assessment would need to be evaluated comprehensively in a designated study. If children with uCP with a larger variability in the degree of sensorimotor skills were to be included, one could expect a corresponding variability in the performance in the active position sense assessment within the uCP group. This is because mild-to-moderately severely affected children with uCP may benefit from ipsilesional neuroplastic reorganization enhancing residual motor abilities, which may result in a better performance in active position sense assessment when compared to a more severely affected uCP group (Inuggi et al., 2018). More research on the performance on this task in children with uCP with varying MACS levels is thus required.

Further, we found that younger TDC tended to perform worse than the older ones and in terms of position sense absolute error and the effect of age on proprioceptive error was significant in this group. This confirms results from previous studies (Bairstow and Laszlo, 1981; Marini et al., 2017) and can be explained by the fact that TDC are continuously developing the ability to utilize proprioceptive feedback throughout adolescence (Goble et al., 2005). Interestingly, while the proprioceptive acuity likely reaches its peak in adulthood, it may then start decreasing again during the process of aging, as it has been shown for passive position sense to decrease with age in older adults (Rinderknecht et al., 2017). In fact, it was only in the passive task where the trend of larger proprioceptive errors for younger children was present in TDC. This is consistent with the findings of Stelmach et al., who found that active proprioception was undifferentiated by age in young adults and elderly participants, while passive proprioception was (Stelmach and Sirica, 1986). They hypothesized that age has less influence on active proprioception due to the additional efferent information (Stelmach and Sirica, 1986; Boisgontier et al., 2012). Another possible explanation could be the higher cognitive processing requirements of the passive assessment, since participants rely only on one source of proprioceptive information from afferent neurons only, as no additional position information is coming through the active movement, and that cognitive processing is not yet fully developed in younger children (Laszlo and Baivstow, 1980; Fuentes and Bastian, 2010). Next to this, we found that the effect of age was not significant in uCP, which is in accordance with the literature as it has been demonstrated that different functions on body level are not influenced by age in these children (Klingels et al., 2012) and might suggest that the maturational process of proprioceptive acuity is disturbed in children with uCP (Riquelme and Montoya, 2010). In case of uCP, the overall severity of sensorimotor impairment is more likely to influence the proprioceptive accuracy than age.

Overall, these results underline the advantage of robotic platforms, such as the ETH MIKE, with actuation and sensing capabilities, where several aspects of proprioception, but also motor or somatosensory function, can be evaluated using one device, providing a detailed characterization of patient's impairment profile, while keeping the set-up time and equipment needed to the minimum.

### Strengths and Limitations

The proposed robotic assessment provides higher sensitivity and objectivity as compared to conventional clinical methods, given the precise and objective sensing capability of the technology-driven solution. Robotic assessments present yet another advantage to the conventional methods, that is potentially higher engagement due to the interactive GUI. This is especially important for children, who typically have a shorter attention span than adults while a high level of concentration is required to assess proprioception (Fortenbaugh et al., 2015). Other robotic platforms have already been tested with children (Marini et al., 2017), but none of them proposed a tailored, children-friendly user interface and were mainly investigating the proximal joints of the upper limb (Kuczynski et al., 2016). Providing the element of personalization (choice of a virtual character) and a gamified element (space travel) could have a motivating effect and lead to an increased enjoyment and willingness of children to participate in studies with the ETH MIKE (Dias et al., 2019). Further, a frequent appearance of an encouraging virtual character and repeated task instruction likely increases attention during the task (Kannass et al., 2010), which could have positively contributed to the satisfying reliability results shown in this study. Verbal feedback from participants supported this hypothesis, as many children (uCP and TDC) found the assessment engaging. However, some found it difficult to stay motivated due to the lack of real-time performance feedback, which is inherent to assessments, where learning effects are to be avoided.

Some limitations of the study need to be considered. The active position sense task would need to be modified for future studies to ensure exact comparability with passive proprioception modality and improve the test administration in children with more impaired hand function. The active task only included two possible positions to which participants needed to move their finger, while passive proprioception, based on previous studies (Rinderknecht et al., 2016; Zbytniewska et al., 2021), consisted of 11 positions to which the finger was moved by the robot in a random order. The second position in flexion (40° from joint neutral position) of the active position sense task should be reduced as it was difficult in the more impaired children with uCP to reach, possibly due to spasticity, limited range of motion or muscle shortage (Klingels et al., 2012). Future development of robotic tasks would also benefit from including an assessment of kinesthesia to build a comprehensive overview of proprioception in uCP, as some distinct properties of kinesthesia and position sense have already been shown in stroke participants (Semrau et al., 2019).

Further, as a limitation to the study design, we only considered discriminant validity, while concurrent validity is frequently addressed and required to comprehensively evaluate validity (Prinsen et al., 2018; Kanzler et al., 2020). For this, it would be beneficial if future work compared results of the robotic tasks to conventional clinical assessments. Previous work in stroke has shown moderate correlations between robotic and clinical assessments of proprioception, likely due to the ceiling effects of clinical scales (Lincoln et al., 1991; Zbytniewska et al., 2021). Those results would need to be confirmed by a designated study in children with uCP, also ideally including neurophysiology and neuroimaging data to fully validate and explain the results. It also needs to be pointed out that only 10 participants in each group (uCP and TDC) were included in this study and a larger cohort would be required to confirm the presented findings, especially with respect to the clinical findings (influence of age on proprioception, relationship between active and passive proprioception). Based on similar studies in uCP (Hung et al., 2004; Reid et al., 2010; Preston et al., 2014) we expected that a sample of 10 children with uCP and 10 TDC would already be sufficient to address the main points of interest of this paper, namely, to show reliability and validity of the outcome measures of passive and active position sense assessments before moving forward with using the platform to answer other clinically relevant questions in uCP. Furthermore, group matching was based solely on age and not on gender, providing no insight into a potential gender effect on position sense. Nonetheless, there is no clear evidence of a gender effect on proprioception in the literature and there are no identified neurophysiology-related reasons for it (Zbytniewska et al., 2021).

Finally, this study only considered the index finger MCP joint. As mentioned earlier, there is evidence of agreement in somatosensory impairments between adjacent body areas after stroke, however, these results would need to be confirmed through a designated study in uCP (Busse and Tyson, 2009). It would be in fact of interest to compare position sense accuracy between fingers, wrist and proximal upper limb, given that technologies focused on each of these levels exist, however, each robotic device implements a different principle of proprioception assessment (Cappello et al., 2015; Kenzie et al., 2017; Zbytniewska et al., 2021). Future work would require implementing a common assessment principle among different robotic platforms for a comprehensive assessment of proprioception of the full upper limb.

## Conclusions

In conclusion, this study successfully showed test-retest reliability and discriminant validity of a robot-assisted assessment of finger active and passive position sense in children with uCP and TDC. The optimization of the tasks for children ensured engagement and motivation. Thanks to the novel method of quantifying proprioception, we gained new insights into differences between position sense modalities, and how they are influenced by hand dominance and age. We found that active position sense was more accurate in TDC. This was not the case for children with uCP, which might be explained by either a dissociation between these two proprioceptive modalities at the level of the brain lesion or by active proprioception assessment being to some extent influenced by the additional motor impairments. We also found that passive position sense was influenced by age in TDC but not in children with uCP, as proprioceptive accuracy may rather be influenced by overall sensorimotor impairment due to brain lesion than age in children with uCP. Overall, the robot-assisted assessment method used in this study provides first, promising insights into the mechanisms of position sense in children with uCP and TDC. Future studies, including larger samples, will aid in further unraveling the impact of proprioceptive deficits in children with uCP.

## Data Availability Statement

Raw data will be made available upon reasonable request.

## Ethics Statement

The studies involving human participants were reviewed and approved by Ethical Committee of the University Hospitals Leuven (S62906). Written informed consent to participate in this study was provided by the participants' legal guardian.

## Author Contributions

Device design and development: MZ-M, CMK, RG, and OL. Study design: LD, LM, EO, KK, and HF. Data collection: LD, LK, and EO. Data analysis and interpretation: MZ-M, CMK, LD, LK, LM, EO, RG, OL, KK, and HF. Manuscript writing: MZ-M, LD, LK, LM, CMK, EO, OL, HF, and KK. All authors read and approved the final manuscript.

## Funding

This work was supported by the Swiss National Science Foundation (project 320030L_170163), the Flemish Research Foundation (FWO project, G0C4919N), and the National Research Foundation, Prime Minister's Office, Singapore under its Campus for Research Excellence and Technological Enterprise (CREATE) program. Open access funding was provided by ETH Zürich.

## Conflict of Interest

The authors declare that the research was conducted in the absence of any commercial or financial relationships that could be construed as a potential conflict of interest.

## Publisher's Note

All claims expressed in this article are solely those of the authors and do not necessarily represent those of their affiliated organizations, or those of the publisher, the editors and the reviewers. Any product that may be evaluated in this article, or claim that may be made by its manufacturer, is not guaranteed or endorsed by the publisher.
