# Supplementary material for: Reliable and Valid Robotic Assessments of Hand Active and Passive Position Sense in Children With Unilateral Cerebral Palsy
Source: Front Hum Neurosci. 2022 Aug 1;16:895080. doi: 10.3389/fnhum.2022.895080 (PMC9376476; doi:10.3389/fnhum.2022.895080)
Supplement: Supplementary file 1 [file Data_Sheet_1.PDF]

Reliable and valid robotic assessments of hand active and passive position sense in children with unilateral cerebral palsy - **supplementary material**

Monika Zbytniewska-Mégret*, Lisa Decraene*, Lisa Mailleux, Lize Kleeren, Christoph M. Kanzler, Roger Gassert, Els Ortibus, Hilde Feys, Olivier Lambercy**, Katrijn Klingels**
*co-first authors, **co-last authors

8th of June 2022

Table SM1: Demographic and clinical data of children with cerebral palsy. Acronyms: MACS: Manual Ability Classification System, PP: Passive Position Sense, AP: Active Position Sense, M: Male, F: Female, y: years, m: months.

ID	MACS	Gender	Age	Test PP (°)	Retest PP (°)	Test AP (°)	Retest AP (°)
Dominant hand							
1	2	M	10y 6m	13.12	15.66	10.62	7.69
2	2	M	12y 3m	16.63	7.99	6.67	3.45
3	1	M	14y 11m	5.98	8.78	11.84	3.86
4	2	F	9y 11m	7.08	9.22	6.36	6.91
5	2	F	7y 5m	8.37	9.67	12.72	8.71
6	1	F	15y 6m	6.84	4.79	8.13	6.79
7	2	M	14y 3m	29.45	20.79	2.82	7.68
8	1	F	12y 11m	9.11	17.78	4.97	5.29
9	1	F	10y 2m	4.68	4.44	3.30	6.06
10	1	F	7y 3m	12.56	16.02	9.73	8.40
Non-dominant hand							
1	2	M	10y 6m	7.03	9.35	5.76	6.19
2	2	M	12y 3m	8.44	8.04	10.31	24.40
3	1	M	14y 11m	7.43	14.15	11.65	9.68
4	2	F	9y 11m	7.72	11.38	24.32	15.31
5	2	F	7y 5m	15.27	14.06	18.33	21.30
6	1	F	15y 6m	8.86	9.11	8.45	11.96
7	2	M	14y 3m	21.06	18.34	15.25	14.98
8	1	F	12y 11m	18.51	11.39	19.37	16.90
9	1	F	10y 2m	5.90	3.78	5.47	6.04
10	1	F	7y 3m	12.93	17.88	6.88	12.24

Table SM2: Demographic and clinical data of typically developing children.

ID	Gender	Age	Test PP (°)	Retest PP (°)	Test AP (°)	Retest AP (°)
Dominant hand						
1	M	9y 6m	14.67	10.07	3.64	4.93
2	F	13y 4m	4.57	6.48	6.21	5.31
3	F	12y 5m	4.07	4.22	3.57	3.29
4	M	12y 5m	5.98	2.29	6.73	6.02
5	M	11y 6m	10.22	8.49	8.32	11.09
6	M	9y 6m	13.50	7.90	5.44	5.04
7	M	11y 9m	5.14	5.84	2.99	5.20
8	F	14y 0m	6.37	4.68	9.47	7.09
9	F	14y 5m	4.02	4.07	2.08	3.07
10	M	7y 1m	20.61	19.82	7.20	5.69
Non-dominant hand						
1	M	9y 6m	9.33	7.14	5.37	7.05
2	F	13y 4m	4.94	3.75	7.67	7.17
3	F	12y 5m	3.19	6.24	3.85	4.86
4	M	12y 5m	7.93	3.34	4.39	4.44
5	M	11y 6m	11.20	3.79	6.18	4.24
6	M	9y 6m	14.79	9.86	3.01	5.75
7	M	11y 9m	4.67	3.61	4.03	3.91
8	F	14y 0m	3.82	4.26	3.37	4.66
9	F	14y 5m	7.27	4.00	1.85	4.16
10	M	7y 1m	10.47	11.83	9.07	6.23
